# Associations of Biotin Levels in Serum and Follicular Fluid With ICSI Success: A Cross‐Sectional Study From Iraq

**DOI:** 10.1155/ogi/8841801

**Published:** 2025-12-28

**Authors:** Zainab Abdul Ameer Jaafar, Nawal Aziz Bakir, Dina Akeel Salman

**Affiliations:** ^1^ Department of Obstetrics and Gynecology, College of Medicine, Al-Mustansiriya University, Baghdad, Iraq, uomustansiriyah.edu.iq; ^2^ Heet General Hospital, Anbar, Iraq

**Keywords:** biotin, follicular fluid, metabolic markers, ovarian response, pregnancy outcomes

## Abstract

**Background:**

Biotin (vitamin B7) has been identified as an essential cofactor within metabolism and gene expression. The purpose of this study is to evaluate the level of biotin within serum and follicular fluid aspirates among infertile women undergoing IVF cycles and to compare these levels with ovulation sensitivity and rates of pregnancy.

**Methods:**

In this observational study with 50 patients with infertility receiving intracytoplasmic sperm injection (ICSI) treatment, women were classified according to ovarian responsiveness to anti‐Mullerian hormone (AMH) levels and the number of retrieved eggs. At the time of egg retrieval, the levels of biotin in the blood and the ovarian fluid were tested. The study maintained strict reporting according to the STROBE criteria.

**Results:**

There was no significant difference with respect to either serum levels or follicular fluids’ biotin among normal responders, poor responders, and hyperresponders. In contrast, there was an inverse correlation between the values of follicular biotin and AMH (*p* = 0.033) and BMI (*p* = 0.022). The data revealed that higher numbers of total and mature oocytes had significant influences on the outcome of pregnancies (*p* = 0.032 and *p* = 0.014), respectively; however, higher values of either serum fluids’ biotin or follicular fluids’ biotin had no significant effect.

**Conclusion:**

Concentrations in serum and follicular fluid of biotin do not play any role in IVF outcome. The negative correlation with AMH and BMI levels might indicate the role of biotin in the ovaries as an indicator of ovarian metabolism. The result provides valuable data in understanding the possible role of biotin in the microenvironment of the ovaries and the need to study it further.

## 1. Introduction

Infertility has been quoted to occur in one out of every six couples in the world [1]. Female factors that lead to infertility are numerous and include ovulation disorders, tube damage, any form of lesions within the vagina and uterus, and endocrine disorders [2]. Recent studies indicate that micronutrients are essential in “reproductive health” and act either singularly or in conjunction with assisted reproductive technologies (ARTs) [3]. Micronutrients such as “antineoplastic factors” (such as antioxidants), B‐complex vitamins, vitamin D3, and essential fatty acids (EFAs), among others, are believed to “improve fertility rates” [4].

Of these, biotin (vitamin B7), one of the water‐soluble B‐vitamins, is pivotal in crucial metabolic processes such as fatty acid synthesis, gluconeogenesis, and amino acid catabolism. Biotin is also crucial in mitochondrial function, gene regulation, and cell proliferation, processes that are essential for follicular development as well as for oocyte competence [5]. Though it has significant biological functions and has been widely distributed in over‐the‐counter fertility vitamins, there has been little research conducted that investigates the use of biotin during human reproduction. The importance of biotin to reproduction in other animals has been shown in many studies carried out on domestic fowls. In these birds, biotin was shown to be crucial to the development of embryos. Evidence from rodents also confirms that biotin deficiency in pregnancy can result in malformations and growth retardation of the offspring, as well as an increase in resorption and embryo loss [6, 7]. In addition, inadequate biotin results in abnormal maturation and quality of the ovum in mice; besides, deficiency during pregnancy is possibly related to preterm labor and growth restriction [8, 9].

Spontaneous biotin deficiency can occur during pregnancy, especially during the third trimester [10]. The reason behind it has been proposed to be higher demand in the mother and lower placental transport of biotin in both humans and animals [10]. In studies conducted on pregnant women, higher 3‐hydroxyisovaleric acid (3HIA) levels have been found in the urine during the initial stages of pregnancy due to mild biotin deficiency [11, 12]. Notably, this metabolic disruption improves with biotin supplementation, implying that current recommended intake levels may be inadequate to meet the increased physiological needs of pregnancy [11, 12].

In addition to metabolism, biotin is believed to contribute to regulating genes through its action on nuclear enzymes such as HLCS that modulate chromatin structure and gene expression. Even though histone biotinylation occurs infrequently, mutations in the HLSC gene are thought to result in genomic instability with implications for the integrity and development of embryos [13, 14].

The purpose of this study is to evaluate the levels of biotin in the sera and follicular fluids of infertile women undergoing IVF treatment and to evaluate the correlation with parameters of ovarian response and the outcome of pregnancy.

## 2. Patients and Methods

This was a cross‐sectional observational study that took place over 10 months, starting from 1st January 2024 to 31st October 2024. The study was carried out in two large infertility centers in Iraq; the Fertility Center in the Al‐Sadder General Teaching Hospital in Al‐Najaf Al‐Ashraf City and the High Institute for Infertility Diagnosis and ARTs in the College of Medicine of Al‐Nahrain University in Al‐Kadhimiya District, Baghdad. Ethical permission was sought from the Ethics Committee in the Scientific Council of the Arab Board for Health Specializations, and verbal consent was obtained from all participants before their inclusion in the study. The study followed the principles set forth in the Strengthening the Reporting of Observational Studies in Epidemiology (STROBE) statement [15].

### 2.1. Study Population and Grouping

The study involved fifty infertile women who had undergone intracytoplasmic sperm injection (ICSI) treatment. The subjects had been placed in three different categories depending on ovarian responsiveness to the process according to anti‐Mullerian hormone (AMH) levels, age, and the number of harvested eggs through the POSEIDON criteria.

The first category comprises 22 women with normal ovarian responders, who are aged 30.18 ± 1.8, with AMH values greater than 1.2 ng/ml, and who had ideal ovarian responsiveness with 9–16 harvested eggs. The second group consisted of 19 women labeled as poor responders (mean age: 36.21 ± 1.7 years), AMH levels below 1.2 ng/mL, and a suboptimal ovarian response, with 4–9 oocytes or ≤ 4 oocytes retrieved. The third group consisted of 9 women diagnosed as hyperresponders (mean age: 26.1 ± 1.1 years), AMH levels over 3.5 ng/mL, and an ovarian response of at least 16 oocytes.

In the study, all women had been diagnosed with either primary or secondary infertility. Data parameters such as age, type and duration of infertility, causes of infertility, treatment history, and IVF attempts had been gathered through the use of a structured questionnaire.

### 2.2. Inclusion and Exclusion Criteria

Women aged 20–42 years, with a body mass index (BMI) between 18 and 35 kg/m^2^, who were nonsmokers and free of systemic or endocrine disorders, were eligible for inclusion. Participants were excluded if they were over 42, current smokers, had systemic or endocrine diseases, were suffering from acute infections, or had taken vitamin supplements, especially biotin, within the three months before the study.

### 2.3. Ovarian Stimulation Protocol

All participants received controlled ovarian hyperstimulation using a gonadotropin‐releasing hormone (GnRH) antagonist protocol. Gonadotropin therapy (Gonal‐F; Serono, Italy) started on the second day of the menstrual cycle, with doses between 150 and 300 IU per day, adjusted according to the patient’s age, BMI, and antral follicle count. Follicular development was monitored through serial transvaginal ultrasonography every 2–3 days. On the seventh day of stimulation, the gonadotropin dosage was adjusted based on serum estradiol (E2) levels and follicular response.

Once at least one follicle reached 14 mm in diameter, daily subcutaneous administration of cetrorelix (0.25 mg; Merck‐Serono, Germany) was initiated and continued until ovulation triggering. Final maturation was triggered with subcutaneous injection of 250 μg of recombinant human chorionic gonadotropin (hCG) (Ovitrelle0; Merck Ltd, UK) after three follicles had reached the diameter of 18 mm. The retrieval was carried out 35–36 h post‐hCG injection.

### 2.4. Biotin Sample Collection and Processing

During oocyte retrieval, blood and follicular fluid samples were obtained for biotin measurement. A 5‐mL venous blood sample was drawn and transferred into coagulation gel tubes. The samples were incubated at 37°C for 30 min, followed by centrifugation at 3000 rpm. The resulting serum was transferred into 5‐mL Eppendorf tubes and stored at −60°C until biochemical analysis.

In the process of obtaining the follicular fluid, 10 mL was aspirated from 18‐ to 24‐mm follicles with the aid of the 17‐gauge oocyte retrieval needle (Wallace, USA). The aspirate was then centrifuged at 3000 rpm to separate the cell components within the follicular fluid; thereafter, the supernatant was aliquoted in an Eppendorf tube and frozen at −60°C.

### 2.5. Fertilization, Embryo Culture, and Luteal Support

The procedure involved the injection of sperm within 1–4 h after the retrieval process. Fertilization was then checked 16–18 h after the process had taken place, and development was observed until Day 3. The embryos′ quality was also tested with the intention to transfer not more than three 6–8 cell stage embryos to the uterine space 48 h after fertilization.

Supportive luteal phase was initiated on the day of embryo transfer with Cyclogest vaginal progesterone suppositories (400 mg, bid; Merck Actives, England), which continued for two weeks. The biochemical confirmation of pregnancy was carried out 14 days after embryo transfer. The confirmation of pregnancy was made by the detection of one or more gestational sacs with one live fetus and its heart activity on a transvaginal scan at 5–6 weeks’ gestation.

### 2.6. Statistical Analysis

We used Statistical Package for the Social Sciences (SPSS) Version 23.0 (IBM Corp., Armonk, NY), supplemented with Microsoft Excel 2010 for data organization. Descriptive statistics, including means and standard errors of the mean (SEM), were calculated for continuous variables, while categorical variables were expressed as frequencies and percentages.

Comparisons were performed with ANOVA one‐way analysis, with subsequent post hoc analysis if required. Comparisons were carried out with independent sample t‐testing. Chi‐square analysis was carried out to compare proportions among categorical variables such as the proportions of pregnant women with outcome groups.

The correlation between levels of biotin and various parameters (age, BMI, AMH, FSH, number of eggs, and rate of fertilization) was determined by Pearson’s correlation coefficient (*r*). A two‐tailed *p* value ≤ 0.05 was taken to be statistically significant in this study.

## 3. Results

### 3.1. Clinical Characteristics and Group Comparisons

Fifty infertile women undergoing IVF were enrolled and classified into three groups: normal responders (*n* = 22), hyperresponders (*n* = 9), and poor responders (*n* = 19). A comparative analysis revealed statistically significant differences among the three groups in terms of age, AMH levels, total oocyte count, Metaphase II (MII) oocytes, number of embryos transferred, and pregnancy outcomes (Table 1).

**Table 1:** Comparison of the clinical parameters between the studied groups.

Parameters	Normal responders (*N* = 22)	Hyper responders (*N* = 9)	Poor responders (*N* = 19)	*p* value
Age (years)	31.36 ± 1.29	26.11 ± 1.17	35.89 ± 1.63	**0.001**
BMI (Kg/m^2^)	23.03 ± 0.72	24.38 ± 1.62	24.38 ± 1.35	0.611
AMH (ng/mL)	2.36 ± 0.24	5.95 ± 0.49	1.04 ± 0.18	**< 0.001**
FSH (mIU/mL)	6.83 ± 0.87	5.21 ± 0.38	14.13 ± 2.27	0.188
Total oocyte count	11.23 ± 0.97	21.22 ± 1.75	5.47 ± 1.08	**< 0.001**
Metaphase II oocytes	9.05 ± 0.78	15.00 ± 1.52	4.00 ± 0.95	**< 0.001**
Fertilization rates	80.76 ± 4.97	76.58 ± 9.20	74.36 ± 8.30	0.775
Transferred embryos	2.45 ± 0.22	1.56 ± 0.71	1.00 ± 0.30	**0.005**
Positive pregnancy rate N (%)	5 (22.7%)	4 (44.4%)	1 (5.3%)	**0.044**

*Note:* significant (*p* ≤ 0.05); not significant (*p* > 0.05). Bold values indicate significant values.

Abbreviations: AMH, anti‐Mullerian hormone; BMI, body mass index; FSH, follicle‐stimulating hormone; SE, standard error.

The mean age was significantly lower in the hyperresponder group and highest among poor responders (*p* = 0.001). The values of AMH ranged substantially; these values are higher in hyperresponders and lower in poor responders (*p* < 0.001). The total number of oocytes and the mature MII oocytes were higher in hyperresponders (*p* < 0.001). The number of embryos transferred was significantly different (*p* = 0.005), with more embryos transferred to normal responders.

The rates of confirmed pregnancy were highest in the hyperresponder group (44.4%) and lowest in poor responders (5.3%), with a statistically significant difference (*p* = 0.044). In contrast, no significant differences were observed regarding BMI, baseline FSH levels, or fertilization rates.

### 3.2. Serum and Follicular Fluid Biotin Levels

Biotin concentrations measured in both serum and follicular fluid did not differ significantly across the response groups. Mean serum biotin levels were comparable between normal, hyperresponders, and poor responders (*p* = 0.226), as were the levels in follicular fluid (*p* = 0.110) (Table 2).

**Table 2:** Comparison of serum and follicular fluids biotin between the studied groups.

Parameters	Normal responders (mean ± SE)	Hyper responders (mean ± SE)	Poor responders (mean ± SE)	*p* value
Serum biotin (ng/mL)	1.27 ± 0.21	0.86 ± 0.16	0.86 ± 0.16	0.226
Follicular fluids biotin (ng/mL)	0.29 ± 0.04	0.24 ± 0.04	0.39 ± 0.05	0.110

*Note:* Not significant (*p* > 0.05).

### 3.3. Correlation Between Biotin and Clinical Parameters

Pearson correlation analysis showed a statistically significant negative correlation between follicular fluid biotin levels and two key clinical parameters: BMI (*p* = 0.022) and AMH (*p* = 0.033). Serum biotin levels were not significantly correlated with any clinical variables, including age, BMI, AMH, FSH, total oocytes, MII oocytes, or fertilization rate (Table 3; Figure 1).

**Table 3:** Correlation between serum and follicular fluids biotin with patient’s clinical parameters.

Parameters	Statistics	Serum biotin	Follicular fluids biotin
Age	*r*	−0.034	0.067
	*P* value	0.816	0.646
BMI	*r*	−0.272	−0.324
	*P* value	0.056	**0.022**
AMH	*r*	0.049	−0.302
	*P* value	0.738	**0.033**
FSH	*r*	0.277	−0.018
	*P* value	0.051	0.904
Total oocyte count	*r*	0.038	−0.157
	*P* value	0.796	0.277
Metaphase II oocytes	*r*	0.097	−0.098
	*P* value	0.505	0.500
Fertilization rate	*r*	0.121	−0.168
	*P* value	0.419	0.258

*Note:* The bold values indicate statistically significant correlations (*p* < 0.05).

Figure 1Correlation between follicular fluid biotin concentrations and clinical parameters. (a) Scatter plot showing the inverse correlation between follicular fluid biotin levels and patients’ body mass index (BMI) (*p* = 0.022). (b) Scatter plot showing the inverse correlation between follicular fluid biotin levels and anti‐Müllerian hormone (AMH) concentrations (*p* = 0.033). Linear regression lines with 95% confidence intervals displayed.

**Figure:**
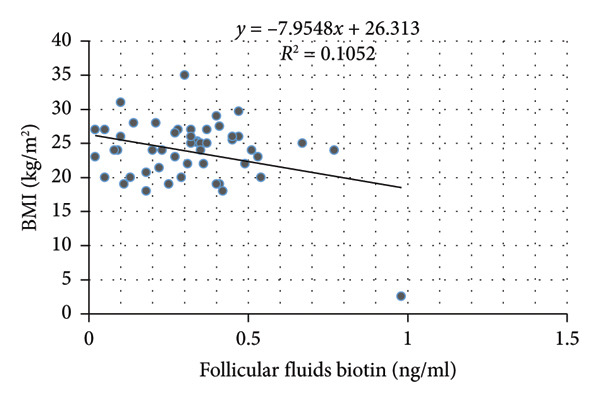
(a)

**Figure:**
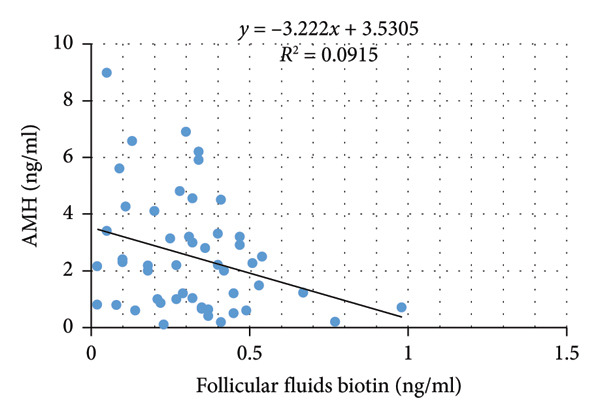
(b)

### 3.4. Pregnancy Outcomes and Biotin Associations

A comparative analysis of clinical parameters between women who conceived (*n* = 10) and those who did not (*n* = 40) demonstrated a significantly greater retrieved oocytes and mature (MII) oocytes in the pregnant group, with *p* values of 0.032 and 0.014, respectively. However, there were no statistically significant differences in age, BMI, AMH, FSH, fertilization rates, or biotin levels (both serum and follicular fluid) between the two groups (Table 4).

**Table 4:** Comparison of clinical parameters between pregnant and nonpregnant females.

Parameters	Pregnant females (*N* = 10)	Nonpregnant females (*N* = 40)	*p* value
Age (years)	28.50 ± 1.63	33.05 ± 1.13	0.064
BMI (Kg/m^2^)	22.40 ± 1.01	24.13 ± 0.78	0.301
AMH (ng/mL)	3.60 ± 0.59	2.23 ± 0.32	0.057
FSH (mIU/mL)	6.62 ± 1.31	9.98 ± 2.58	0.524
Total oocyte count	15.20 ± 1.57	9.75 ± 1.16	**0.032**
Metaphase II oocytes	12.00 ± 1.18	7.25 ± 0.88	**0.014**
Fertilization rates	76.39 ± 5.86	78.16 ± 4.85	0.859
Serum biotin (ng/mL)	0.72 ± 0.14	1.12 ± 0.14	0.181
Follicular fluids biotin (ng/mL)	0.26 ± 0.04	0.33 ± 0.03	0.289

*Note:* significant (*p* ≤ 0.05); nonsignificant (*p* > 0.05). Bold values indicate significant values.

Abbreviations: AMH, anti‐Mullerian hormone; BMI, body mass index; FSH, follicle‐stimulating hormone.

## 4. Discussion

The most significant findings of this study were the inverse correlations between follicular fluid biotin levels and both BMI and AMH, indicating that lower follicular biotin concentrations were associated with higher ovarian reserve and higher BMI. These might imply the involvement of biotin in the metabolism that occurs in the microenvironment surrounding the follicles, in which higher activity in the ovaries might result in greater use or redistribution with regard to biotin. Of interest is the lack of significant correlation between serum biotin and any parameter of reproduction and hormones.

Despite biotin’s well‐documented biological roles in cellular metabolism, mitochondrial function, and gene regulation, no differences were observed among normal, poor, and hyperresponders, nor between pregnant and nonpregnant patients. This suggests that, in biotin‐replete individuals, biotin concentration alone may not distinguish between varying degrees of ovarian responsiveness or directly influence clinical pregnancy outcomes.

To some extent, our results are in correlation with those found by Yanagihara et al. (2022) [16], who analyzed the levels of biotin in the sera and follicular fluids of IVF/ICSI patients and found that there is a significant correlation between the two. Though no significant difference was found in the trend toward higher biotin values in pregnant women compared to that in nonpregnant women, similar to our study; however, their result that “biotin fails to affect the quality of oocytes and the rates of conception in biotin‐sufficient subjects” holds true with that found in this study. The study carried out by Al‐Mohammedy et al. (2024) [17] found that “biotin supplementation has no effect on sex hormones such as estradiol‐17‐β and progesterone” in Iraqi ewes during pregnancy; though carried out on models other than medicine but adds to the evidence that though biotin has significant metabolic activity in the body, it has no effect on the steroid secretions within the ovaries during conception.

The developmental influence of biotin observed in our study is supported by experimental findings from Alkubaisy et al. (2021) [18], who showed that in‐ovo biotin injection significantly improved embryonic growth, hatchability, and decreased embryonic mortality in chick embryos. Their results emphasize biotin’s essential role in early cellular development and metabolic regulation. Although conducted using an avian model, their findings suggest that biotin availability during key developmental periods may affect reproductive outcomes, reinforcing our rationale for studying biotin levels in the human follicular environment during IVF. Supporting this, Salian et al. (2019) [19] demonstrated that biotin supplementation in sperm preparation media improved sperm motility, fertilization potential, and preimplantation embryo development in a mouse model. Their results, showing better blastocyst quality and higher hatching rates, strengthen the biological plausibility of biotin’s role in early reproductive events. Although our study did not find a significant link between biotin levels and pregnancy outcomes, these experimental results suggest that biotin may influence embryo competence through mechanisms not solely reflected by follicular or serum measurements. Sidibe et al. (2007) [20] brought forth a novel proteomics procedure that utilizes biotin to study membrane and membrane‐binding proteins in smooth muscle cells generated from embryonic stem cells. The proteins identified in this study are essential to cell surface functions with implications in cell adhesion and development.

In light of this, apart from the abovementioned factors, our analysis has further supported the predicted tendencies in the study of reproductive physiology. The values of AMH and produced oocytes were higher in hyperresponders, and those who had poor responses had lower values in the ovarian parameters. The pregnant group had higher values in the total and MII values of produced oocytes; this further proved that the numbers and maturity of produced oocytes are pertinent predictors in IVF treatments. Li et al. (2017) [21] demonstrated that biotin supplementation at common over‐the‐counter doses (10 mg/day) significantly interfered with several hormone assays, including those for TSH and parathyroid hormone, leading to falsely elevated or suppressed values. While our study did not observe hormonal fluctuations associated with serum or follicular biotin levels, these findings highlight the complexity of biotin’s interactions with hormonal systems and the need to interpret both endocrine and fertility‐related outcomes with caution in the context of biotin exposure.

The current results that there was no direct correlation between blood and follicular fluid levels of biotin and the outcome of the pregnancy are also supported by other observations in women with genetic disorders of biotin metabolism. Indeed, recently, there was one woman with holocarboxylase synthetase deficiency who had successfully carried her pregnancy to term without any obstacles to her metabolism while taking 100 mg daily doses of biotin [22]. This helps to confirm that if there is sufficient biotin present, there are no concerns about metabolism to impede reproduction; there is no direct correlation between blood levels and rates of IVF success and fertility.

In summary, our data suggest that follicular fluid biotin may reflect ovarian metabolic activity rather than serve as a direct biomarker of IVF success. The observed correlations with AMH and BMI warrant further investigation, particularly in populations with known micronutrient imbalances or metabolic disorders.

### 4.1. Limitations

There are some limitations found in this study. First, the study had a smaller sample population, which may impact its sensitivity to recognize the subtle correlation that might exist between the levels of biotin and IVF results. The study was also observational; therefore, there are no hypotheses that can be made to confirm the existence of a clear correlation between the levels of biotin and the results shown in the parameters of reproduction. The study also does consider the effect that assay disruptions caused by hormones might pose to biotin.

## 5. Conclusion

Although serum and follicular fluid biotin levels did not significantly differ across ovarian response groups or between pregnant and nonpregnant patients, an inverse correlation was observed between follicular biotin levels and both AMH and BMI. These findings suggest that follicular biotin may reflect local ovarian metabolic activity rather than serve as a direct biomarker of IVF success. Further research with larger cohorts is warranted to explore the mechanistic pathways through which biotin may influence follicular dynamics and reproductive outcomes.

## Ethics Statement

The approval to conduct this study was obtained from the Scientific and Ethical Committee in the College of Medicine at Al‐Mustansiriyah University. The protocol was prepared to make sure that it met the requirements related to national legislation and ethical standards that should be adopted in research involving human subjects. The approval was sought within the time frame allotted in the project (IRB Approval number: 280; approval date: January 2024).

## Consent

Informed consent was sought from each participating subject. The principles outlined in the Declaration of Helsinki were adopted.

## Conflicts of Interest

The authors declare no conflicts of interest.

## Author Contributions

Zainab Abdul Ameer Jaafar: conceptualization, methodology, data collection, drafting original draft, and final approval. Nawal Aziz Bakir: literature review, data analysis, paper write‐up, and critical paper revision. Dina Akeel Salman: data curation, statistical analysis, literature review, and manuscript editing.

## Funding

No funding was received for this study.

## Supporting Information

The supplementary material associated with this article includes the “Biotin STROBE_checklist.docx,” which provides a detailed checklist confirming adherence to the STROBE guidelines. It outlines the reporting quality and transparency of the current cross‐sectional study.

## Supporting information


**Supporting Information** Additional supporting information can be found online in the Supporting Information section.

## Data Availability

The data can be obtained from the corresponding author.
